# Poly(ADP-Ribose) Polymerase Inhibitor PJ34 Reduces Brain Damage after Stroke in the Neonatal Mouse Brain

**DOI:** 10.3390/cimb43010025

**Published:** 2021-06-07

**Authors:** Philippe Bonnin, Tania Vitalis, Leslie Schwendimann, Alexandre Boutigny, Nassim Mohamedi, Valérie C Besson, Christiane Charriaut-Marlangue

**Affiliations:** 1 U1148, LVTS, INSERM, F-75018, Physiologie Clinique—Explorations Fonctionnelles, Hôpital Lariboisiere, Université de Paris, 75010 Paris, France; philippe.bonnin@aphp.fr (P.B.); alexandre.boutigny@gmail.com (A.B.); nassim.mohamedi@aphp.fr (N.M.); 2 INSERM, Hôpital Robert Debré, Université de Paris, U1141 NeuroDiderot, 75019 Paris, France; tnvitalis@gmail.com (T.V.); Leslie.Schwendimann@inserm.fr (L.S.); 3 UMR-S1144—Optimisation Thérapeutique en Neuropsychopharmacologie, Faculté de Pharmacie de Paris, Université de Paris, 75006 Paris, France; valerie.besson@parisdescartes.fr

**Keywords:** neonatal ischemia, doppler ultrasonography, collateral circulation, blood-brain barrier, somatostatin receptor, octreotide, astrocytes

## Abstract

The poly(ADP-ribose) polymerase inhibitor PJ34 has recently been reported to increase cerebral blood flow, via the endothelial NO synthase, in the naive mouse brain throughout life. We addressed here the benefits of PJ34 after neonatal ischemia on hemodynamics and components of the neurovascular unit including the blood-brain barrier (BBB), microglia, and astrocytes. Nine-day-old mice were subjected to permanent MCA occlusion (pMCAo), and treated with either PBS or PJ34 (10 mg/kg). Mean blood-flow velocities (mBFV) were measured in both internal carotid arteries (ICA) and basilar trunk (BT) using Doppler-ultrasonography. BBB opening was assessed through somatostatin-receptor type-2 internalization and immunohistochemistry at 24 and 48 h. Lesion areas were measured 8 days after ischemia. In PBS-treated mice, pMCAo involved a drop in mBFV in the left ICA (*p* < 0.001 vs. basal), whereas mBFV remained stable in both right ICA and BT. PJ34 prevented this drop in the left ICA (*NS* vs. basal) and increased mBFV in the right ICA (*p* = 0.0038 vs. basal). No modification was observed in the BT. In contrast to PBS, BBB disruption extent and astrocyte demise were reduced in PJ34 mice only in the rostral brain at 48 h and 8 days post-pMCAo, respectively. Accordingly, 8 days after pMCAo, affected areas were reduced in the rostral brain (Bregma +0.86 and +0.14 mm), whereas total tissue loss was not reduced after PJ34 (4.0 ± 3.1%) vs. PBS (5.8 ± 3.4%). These results show that PJ34 reduced BBB permeability, astrocyte demise, and tissue loss (particularly in the rostral territories), suggesting that collateral supply mainly proceeds from the anterior ICA’s branches in the ischemic neonatal mouse brain.

## 1. Introduction

Neonatal arterial stroke is a cerebrovascular event that occurs near the time of birth, producing significant morbidity and severe long-term neurological and cognitive deficits such as cerebral palsy, impaired vision and language function, and epilepsy. Currently, there is no specific treatment for neonatal stroke except supportive care including the management of seizures [1].

Neonatal stroke is a disorder affecting both macro- and micro-vascular networks during ischemia and reperfusion, leading to blood-brain barrier (BBB) disruption, edema, and cell death. Poly(ADP-ribose) polymerase (PARP) inhibition has been demonstrated to reduce cerebral infarct lesion in neonatal rats [2] and adult mice [3].

In the peripheral vascular system, the endothelial dysfunction consecutive to diabetes mellitus has been related to an endothelial depletion of NADPH, an essential cofactor of the endothelial nitric oxide (NO) synthase (eNOS) [4]. Pharmacological inhibition of PARP with PJ34 (10 mg/kg) can prevent endothelial dysfunction associated with hypertension and aging [5] or induced by endothelin-1 [6]. In the cerebral vascular system, we recently reported that a single dose of the PARP inhibitor PJ34 (10 mg/kg) is able to increase cerebral blood-flow (CBF) by recruitment of the microvascular vasodilation reserve (without sex effect), and is likely to act on the endothelial function throughout life (with a major effect in the neonatal and adult brain) in the naive mouse [7].

In the brain, the neurovascular unit (NVU) allows the relationship between brain parenchyma and its micro-vessels. The blood-brain barrier (BBB) is an essential part of the NVU, which undergoes disruption in cell-cell communication between endothelial cells, pericytes and astrocytes early after stroke [8]. How reperfusion-reoxygenation may affect BBB function remains still less evaluated in the immature brain, depending on amplitude and duration of CBF reduction, and on the model used as vascular differences occurred in rodents [9].

In this study, we address the cerebral vasodilator property of PJ34 (given as a single dose) after neonatal ischemia, and evaluate its incidence on blood-flow rerouting and NVU actors (early BBB leakage using G protein coupled receptor internalization [10]), microglia, and astrocyte phenotypes as indicators of tissue damage.

## 2. Results

### 2.1. Collateral Recruitment by PJ34 after Neonatal Ischemia

**Figure 1:**
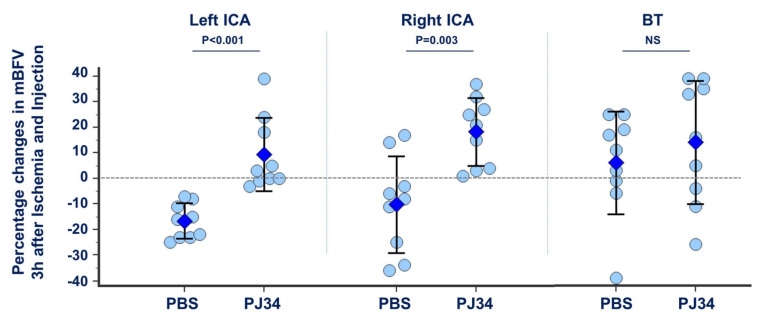
Relative changes in mBFV recorded in the three afferent arteries of the circle of Willis 3 h after ischemia and treatment. pMCAo produced an amputation of the vascular network, then the left ICA showed a drop in mBFV in PBS-treated mice. PJ34-treated mice did not show this drop; this was evocative of the establishment of the collateral supply through the cortical anastomoses between neighboring cerebral territories. In the right ICA, mBFV remained stable after ischemia, whereas PJ34 produced increased mBFV in both blood rerouting through the circle of Willis and then the cortical anastomoses. In the BT, no significant modification was recorded in PBS- and PJ34-treated mice (not significant—NS; *n* = 9 mice per group).

No differences in mBFV were recorded in the three arteries of the circle of Willis before ischemia and injections between PBS- and PJ34-treated mice. Three hours after ischemia, mBFV in the left ICA decreased in PBS-treated mice (−17 ± 7%, *p* = 0.0003, pMCAo vs. basal), representative of the exclusion of the vascular network downstream from the site of electrocoagulation. Conversely, in PJ34-treated mice, mBFV remained stable (NS, pMCAo vs. basal; *p* < 0.001, PJ34- vs. PBS-treated). In the right ICA, PBS-treated mice exhibit on average no modification in mBFV after pMCAo. Nevertheless, distribution of the mBFV was scattered over a large range in the right ICA: two mouse pups presented increased mBFV (+14% and +17%) and two presented decreased mBFV (−36% and −34%). These individual variations were not related to modifications in the heart rates, thus in the cardiac output nor in mBFV in the left ICA. The large distribution of the individual values could then be due to individual difference in the time-delay for establishment of the blood-flow rerouting through the anterior anastomosis of the circle of Willis. In contrast, under PJ34, mBFV in the right ICA increased (+18 ± 13%, *p* = 0.0038, pMCAo vs. basal, *p* = 0.0026, PJ34- vs. PBS-treated) (Figure 1, Supplementary Materials Table S1). Heart rates and mBFV in the BT remained stable after pMCAo in both groups.

### 2.2. PJ34 Reduces BBB Permeability in the Anterior Brain

**Figure 2:**
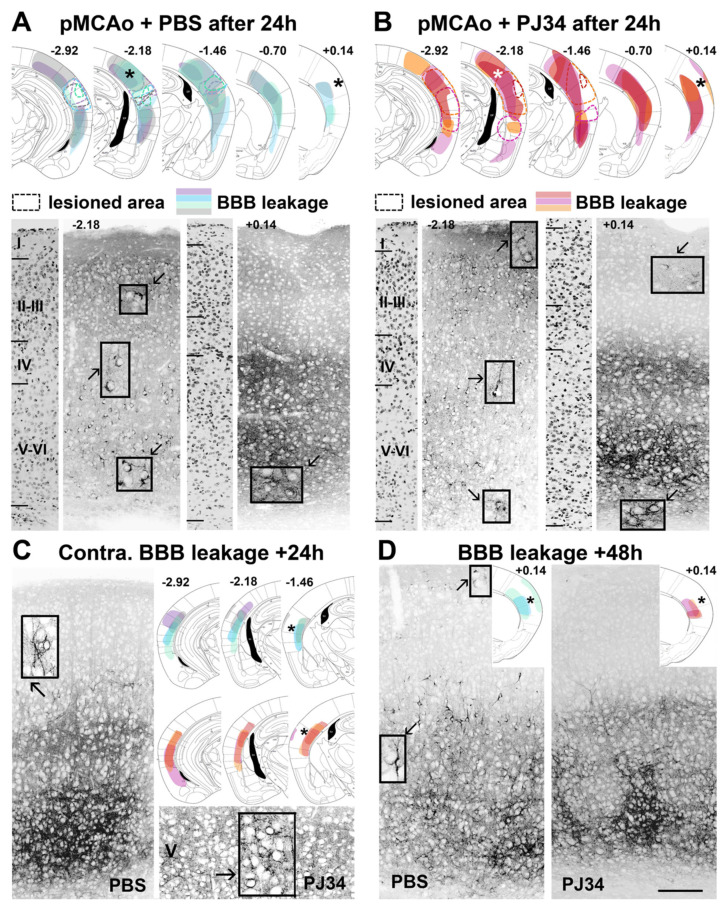
Blood-brain barrier permeability extent in PBS- and PJ34-treated mice following pMCAo. (**A**,**B**): Cartographies at 24 h (bregma −2.92, −2.18, −1.46, −0.70 and +0.14 mm) of the lesioned areas (doted lines) and regions of SST2 internalization (colorization, one color/animal), and illustrations of SST2 internalization (*star for captions) in PBS- (*n* = 4) and PJ34-treated (*n* = 3) mice. (**A**): At −2.18 mm, SST2 internalization is detected in all cortical layers. Inserted boxes show high magnifications of cells displaying an SST2 labeling concentrated in the somatic (trans-Golgi network) compartment. Alongside, at +0.14 mm, only a few SST2^+^-cells were located in the deep layers V–VI. (**B**): At −2.18 mm in PJ34-, the distribution of SST2^+^ cells in all cortical layers is similar to that observed in PBS-treated mice. At +0.14 mm, SST2 internalization is mainly detected in deep layers V–VI with a few cells in layer I. (**C**): In both PBS- and PJ34-treated mice, SST2 internalization (upper layer V) is only detected at bregma −2.92 to −1.46 mm, contralateral to the lesion. (**D**): At 48 h after pMCAo and at the rostral level +0.14 mm, SST2 internalization is observed in almost all cortical layers in PBS-treated mice (*n* = 3), whereas only deep layers V–VI contain SST2^+^-cells in PJ34-treated mice (*n* = 4). Black star indicates the absence of SST2 labeling. Scale bar represents 125 µm and 30 µm for the inserted captions, respectively.

**Figure 3:**
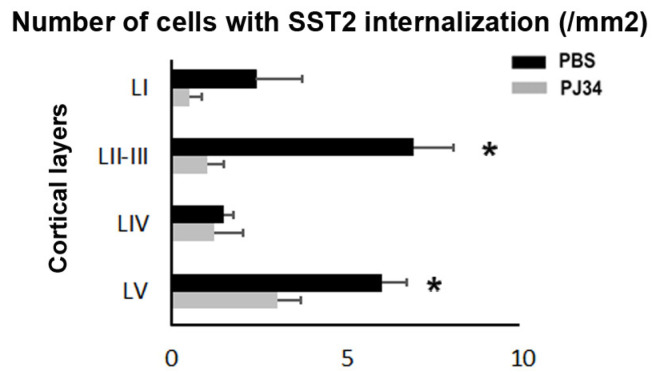
PBS-treated P7 mice show increased numbers of cells showing SST2 internalization in the rostral cerebral cortex 48 h after PMCAo compared to PJ34-treated mice. The graph is showing the quantifications (mean ± SEM) of the densities of cells in the different cortical layers in PBS- (*n* = 3) and PJ34-treated (*n* = 4) pups at the +0.14 bregma level. A single confocal Z plane (20×) was used for the quantification. Note that, due to the very high density of the SST2 staining in layer VI, punctate corresponding to SST2 internalization was difficult to attribute to a specific single cell. Layer VI counting is therefore not reported in the graph. Asterisk is indicated for * *p* values < 0.05.

SST2 internalization, signing BBB integrity defects (Supplementary Materials Figure S1), was observed in the ipsilateral cortex of PBS- and PJ34-treated mice 24 h after pMCAo in layers I–VI between bregma −2.92 to −1.46 mm, but largely spared layers I–IV between bregma −0.70 to +0.14 mm (Figure 2A,B). On the contralateral side, SST2 internalization was transiently observed in deeper caudal cortical layers at 24 h (Figure 2C). Ipsilaterally, SST2 internalization at 48 h was reduced in the rostral brain (bregma +0.14 mm) after PJ34 treatment, and more particularly in layers II, III, and V with an absence of labeling in the superficial layers as compared to PBS-treated mice (Figure 2D and Figure 3).

### 2.3. PJ34 and Microglia in the Anterior and Posterior Brain

In brain parenchyma, microglia cells comprise about 10–15% of all glial cells, display a small body, and are highly ramified [11,12]. After ischemia, microglia are considered conductors of the inflammatory response and a high abundance of activated microglia/macrophages was detected in the penumbra as previously reported [13,14].

**Figure 4:**
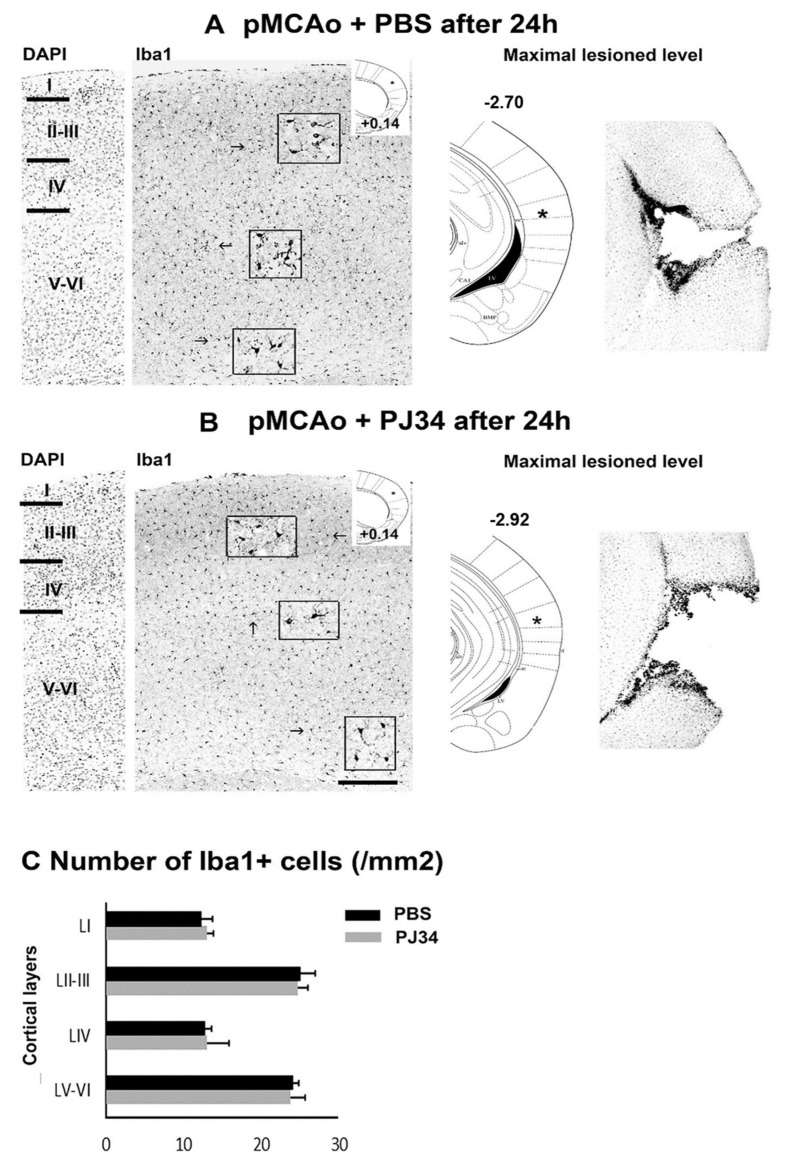
PBS- or PJ34-treated P7 mice show similar numbers of Iba1 expressing microglial cells in the rostral cerebral cortex 24 h after PMCAo. (**A**,**B**): Example of a PBS- (**A**) and PJ34-treated (**B**) pup showing Iba1+ cells in the different cortical layers. Left caption is taken at bregma level +0.14 mm (asterisk in the caption taken from the atlas). Higher magnifications of Iba1+ cells in various cortical layers are presented in the insets. DAPI labeling of the same section is used to annotate the cortical layers. The right panels are showing Iba1-labeling taken at the level where the lesion appears maximal (asterisk; bregma level −2.70 (**A**) and −2.92 (**B**) mm, respectively). Black star indicates the site of the lesion (cavity). (**C**): Quantifications (mean ± SEM) of the densities of Iba1+ cells in the different cortical layers (LI = layer I) in PBS- (*n* = 3) and PJ34-treated (*n* = 4) pups at +0.14 mm of bregma. A single confocal Z plane (20×) was used for the quantification. Scale bar: Left panels 400 microns; right panels 1300 microns.

Numerous Iba-1-positive cells with both ramified and/or ameboid morphology were observed in all ipsilateral cortical layers of PBS- and PJ34-treated mice 24 h after pMCAo (Figure 4A,B). Their number was not different between PBS and PJ34 treatment at the rostral level (+0.14 mm). At the caudal level (−2.70 and −2.92 mm, respectively), a great number of Iba-1+-macrophages was observed around the lesion (Figure 4C).

### 2.4. PJ34 Reduces Tissue Loss and Astrocyte Demise in the Anterior Brain

**Figure 5:**
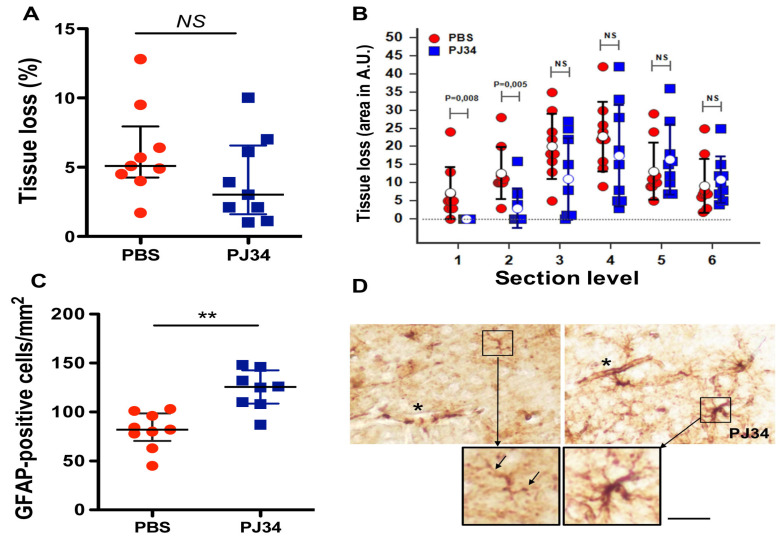
Tissue loss and astrocyte features eight days after ischemia were measured on the same mice used for the measurements of mBFV. (**A**): Total tissue loss in PBS- and PJ34-treated mice. (**B**): Tissue loss measured on six different brain levels (bregma 0.86 (1), 0.14 (2), −0.80 (3), −1.50 (4), −2.30 (5), and −2.92 (6) mm) from rostral to caudal brain. Whereas total tissue loss was not significantly reduced, a significantly reduced area of tissue loss was observed at the level of the two first sections (anterior striatum). Open circles represent the mean ± SD (black, PBS; blue, PJ34). NS = non-significant. (**C**): Quantification of GFAP-positive astrocytes in the cortex (bregma +0.14 mm) in PBS (red) and PJ34-treated (blue) mice. Data are plotted as dots with median and interquartile range, ** *p* < 0.01. (**D**): Representative images of astrocyte morphology in a PBS-treated and PJ34-treated mouse showing loss (in PBS) and presence of GFAP-stained astrocytes endfeet (black star) respectively, and a few astrocytes with clamatodendrotic features (black arrows in enlarged panel). In a PJ34-treated mouse, stellar GFAP-stained astrocytes with more or less thick processes (enlarged panel) were observed. Bar represents 20 µm.

Ipsilateral tissue loss in both groups was preferentially caudal, low, and similar eight days after ischemia (PBS, 5.8 ± 3.4%; PJ34, 4.0 ± 3.1%, *NS*). However, rostral brain sections (bregma +0.86 and +0.14 mm) showed significant reduced area lesions (Figure 5A,B). In addition, and as observed above (see Figure 3), PJ34 trended to shift the lesion towards the caudal side (from bregma −2.70 to −2.92 mm, respectively).

At this time point of recovery, and in the rostral brain section (bregma +0.14 mm), the number of GFAP-positive astrocytes decreased after ischemia (PBS-treated mice), and significantly increased with PJ34 treatment (*p* < 0.01 PJ34 vs. PBS; Figure 5C). In the cortex of naive P17 mice, stellar fibrillary GFAP-positive astrocytes are mainly shown with their endfoot processes in the subpial cortex [15]. In the ischemic lateral cortex (PBS-treated mice), the few remaining astrocytes have lost their distal processes (i.e., undergo clasmatodendrosis [15]) to form GFAP-positive dots (black arrows in Figure 5D). In PJ34-treated mice, astrocytes again displayed a stellar morphology with GFAP immunoreactivity in the processes (Figure 5D).

In the caudal sections (bregma −1.50 to −2.92 mm), reactive GFAP-positive astrocytes are observed around the lesion that formed a dense network both in PBS- and PJ34-treated mice (Supplementary Materials Figure S2).

## 3. Discussion

Our data indicated that PJ34 was able to increase CBF after ischemia in neonatal mice, as previously described [7]. Fortunately, early establishment of this collateral supply reduced BBB permeability and tissue damage in the rostral brain only.

**Figure 6:**
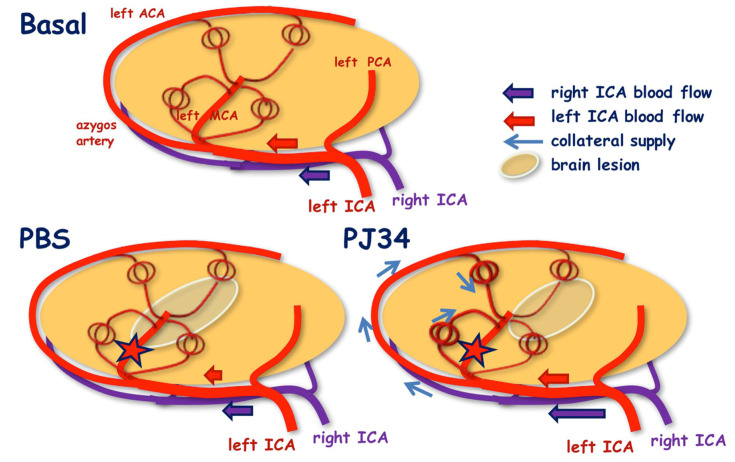
Schematic representation summarizing blood flow re-routing after pMCAo and treatment. After PBS, blood flow is reduced in the left ICA with no collateral flow. After PJ34, blood flow remained stable in the left ICA and increased in the right ICA, leading to collateral supply via the azygos artery. Red star represents the location of pMCAo, i.e., after the first side branches of the MCA.

Enhancing collateral blood supply during the acute phase of ischemia could limit the extension of the core infarct and the degree of disability [16]. The absence of any drop in mBFV in the left ICA in PJ34-treated mice was illustrative of a collateral supply towards the cortical anastomoses extended between the arterial territories of the collateral branches coming from the proximal MCA, upstream the site of pMCAo, and the collateral branches coming from the distal MCA downstream the site of pMCAo. In the right ICA, PJ34 produced an increase in mBFV following the rerouting of collateral blood supply through the proximal right ACA and the azygos artery towards the distal left ACA (Figure 6). This CBF rerouting permitted the supply to the open cortical anastomoses extended between the left ACA and left MCA arterial networks, a blood-flow rerouting already observed after pMCAo in the adult mouse [17]. Nevertheless, in pup mice, the BT was not involved in the early establishment of the collateral supply towards the ischemic territories as previously observed in adults [17].

Permanent MCAo resulted in obvious BBB leakage around the cortical lesion at 24 h, whereas tMCAo did not expose to a BBB patency as attested by the absence of 3 kDa dextran leakage in P7 rats [18]. A transient leakage of small tracers, sucrose, and inulin was observed after hypoxia-ischemia in P9 mice, peaking at 6 h and then progressively resolving over time [19]. Herein, the transient BBB patency in the rostral brain observed in layer I at 24 h only, and not later, may be explained by the early and beneficial recruitment of a collateral blood-flow supply mediated by PJ34, avoiding a deep and extended local ischemia. We also expect that the large cortical BBB leakage at the two dorsal and ventral ends observed at 24 h may be in part transient (and reversible) because the final lesion was relatively small 8 days after ischemia. In addition, BBB disruption was also transiently shown in the contralateral hemisphere, contemporary with a transient and aborted apoptosis [20].

A prominent aspect of inflammatory responses after ischemia is activation of microglial cells. Microglia are the resident macrophages of the central nervous system. Microglia in a normal brain have a highly branched, ‘ramified’ morphology that can rapidly transform into an activated-ameboid morphology in response to a stroke [21]. In our study, a single dose of PJ34 at the reperfusion was not sufficient to reduce microglia activation in the rostral and caudal sections of the brain, and interestingly, longer PJ34 intervention during reperfusion (during 7 days) was associated with more apparent protective effects after cerebral ischemia in adult rats [22]. Two doses of PJ34 (at 24 and 72 h post-ischemia) were shown to increase M2 and M-trans microglia phenotypes in the amygdala associated with an improvement in motor coordination and learning, but without a reduced lesion size [14] suggesting that PJ34 dose regimen in the ischemic mouse pup should be evaluated more deeply.

The presence of clasmatodendrotic astrocytes in the cortical infarct eight days after ischemia suggests that tissue damage is still in progress as previously reported in the neonatal ischemic rat [23], and PJ34 reverses this effect at the rostral extremity. In primary astrocyte cultures submitted to oxygen-glucose deprivation and re-oxygenation, PJ34 was associated with protective effects [24], and combined targeting of either PARP-1/PARP-2 or PARP-12/PARP-3 inhibition after *s. aureus* injury attenuated astrocyte inflammatory responses more effectively compared to knock-down of either PARP alone [25].

In the adult brain, PJ34 (given in two doses) was able to reduce the hemorrhagic transformation induced by recombinant tissue plasminogen activator (rt-PA) after focal ischemia in the mouse [26,27]. The anti-hemorrhagic effect of PJ34 and the finding that rt-PA increases the activation of the PARP enzyme after cerebral ischemia suggest that PJ34 could be used as an adjunctive treatment to improve the safety of thrombolysis in acute ischemic stroke. Furthermore, PARP inhibitors, which appear to be safe for use in humans given the good tolerance of several inhibitors currently in phase I or phase II clinical trials may be given early after stroke. However, in the developing brain hemorrhagic transformation was not evidenced and such clinical trials not evaluated.

Altogether, these data demonstrated that PJ34 may have a pleiotropic action, but with a different timescale between the different NVU components (BBB, astrocytes and microglia), suggesting that PJ34 dose regimen should be adapted to the NVU components responses towards neonatal ischemia in the mouse brain, and to the specific cerebral area studied (cortex, striatum, deep grey nuclei).

## 4. Materials and Methods

### 4.1. Ethic Statement

All experiments were performed in accordance with the European Community guidelines (Directive 2010/63/EU) and the French National guidelines for the care and use of laboratory animals. All animal procedures were approved by the local Ethics Committee in Animal Experimentation (protocol number APAFiS # 03560.02; 19 January 2018), and in compliance with the ARRIVE guidelines (https://www.nc3rs.org.uk/arrive-guidelines, accessed on 12 March 2021).

### 4.2. Neonatal Cerebral Ischemia

WT 9-day-old C57BL6/J mice were purchased from Janvier Labs (Le Genest- St Isle, City, France; *n* = 32, both sexes). Permanent distal middle cerebral artery (MCA) occlusion (pMCAo) was performed under isoflurane anesthesia in 30% O2 and 70% N2O. Immediately after ischemia, mice received either PBS or PJ34 (10 mg/kg i.p.) in a single injection. All animals were included (no death). All experiments were performed in a blinded and randomized manner.

### 4.3. Ultrasound Imaging

Thermoregulated pup mice (*n* = 9 per group) were subjected to ultrasound measurements under isoflurane anesthesia (0.5%) using an echocardiograph (ACUSSON S3000, Siemens, Erlangen, Germany) equipped with a 14.5-MHz linear transducer. The Doppler sample was placed successively in the left (lICA) and right (rICA) internal carotid arteries upstream the posterior, middle and anterior cerebral arteries, then in the basilar trunk (BT) downstream the confluence of both vertebral arteries for Doppler velocity waveform recordings. Time-average mean blood flow velocities (mBFVs) were then measured in each ICA and in the BT just before and 3 h after the pMCAo procedure in the PBB- and PJ34-treated mice. The mBFV measurements were previously investigated in control mice at basal states for intra-observer repeatability. Two series of measurements separated by a 10 min interval were recorded. The repeatability coefficient (RC) was calculated as defined by the British Standard Institution (1979), i.e., according to the formula RC^2^ = ∑Di^2^/n, where Di is the relative (positive or negative) difference within each pair of measurements, and n is the sample. The intra-observer-RC values were 0.3, 0.4, and 0.4 cm/s for left ICA, right ICA, and BT, respectively, largely inferior to the differences exhibited between the different groups when considered as statistically significant. Heart rates were measured and reflected changes in cardiac output, as ventricular volume is quite invariable in newborns.

### 4.4. Tissue Preparation and Blood-brain Barrier Disruption

Mice pups were sacrificed at 24 (*n* = 3–4 each group) and 48 (*n* = 3–4 each group) hours after ischemia. Forty-five mins after intraperitoneal octreotide injection (OCT, 2.5 mg/kg/i.p. diluted in PBS; Sigma-Aldrich, St. Louis, MO, USA), animals were deeply anaesthetized with sodium pentobarbital (150 mg/kg i.p.; Ceva Sante Animal, Libourne, France) and perfused through the ascending aorta with 100 mL of 4% paraformaldehyde (PFA) in 0.1 M phosphate buffer, pH 7.4 (PB). Brains were dissected, post-fixed in the same fixative overnight at 4 °C, cryoprotected, frozen in liquid isopentane at 45 °C and sectioned in the coronal plane at a thickness of 40 µm and collected in PBS. The endogenous SST2 receptor was immunolocalized on brain sections using a rabbit monoclonal anti-SST2 antibody (1:1000, ab134145, Abcam, Cambridge, UK), and visualized with fluorescent secondary antibodies (Alexa Fluor 488, 1:200, Life technologies, Molecular probes, Eugene, OR, USA). Briefly, sections were preincubated in PBS with 5% normal donkey serum (NDS; Sigma-Aldrich, St. Louis, MO, USA) and 0.3% Triton X-100 for 30 min at RT, incubated in anti-SST2 antibody diluted in PBS with 1% NDS and 0.3% Triton X-100 overnight at RT, rinsed in PBS, and incubated in Alexa Fluor 488 (A488)-conjugated donkey anti-rabbit antibody diluted in PBS with 3% NDS and 0.3% Triton X-100 for 1 h at RT. Finally, sections were rinsed in PBS, stained with DAPI 1:1000 in PBS for 2 min at RT, rinsed in PBS, mounted on glass slides and coverslipped with Fluoromount (SouthernBiotech, Birmingham, AL, USA) for confocal microscopic analysis. Area lesions were plotted at the level of the six selected sections from anterior striatum to posterior hippocampus. Microglia/macrophages were stained using rabbit anti-Iba1 (RRID: AB_839504) antibodies and revealed using secondary goat anti-rabbit cyanine 3-coupled antibodies (Jackson Immuno-Research laboratories, West grove, PA, USA), and processed as above.

Lesioned areas and regions showing SST2 internalization in cells were delineated at the level of the five selected sections from bregma level −2.92 mm (posterior hippocampus) to bregma level +0.14 mm (anterior striatum and anterior commissure).

For confocal microscopy, immunofluorescent sections were analyzed using a Leica TCS SP8 confocal scanning system (Leica Microsystems, Wetzlar, Germany). Eight-bit digital images were collected from a single optical plane using a 20 × HC PL APO CS2 oil-immersion Leica objective or a 63 × HC PL APO CS2 oil-immersion Leica objective. Images were inverted in photoshop to obtain black and white negative pictures and were equally adjusted for brightness and contrast. Composite illustrations were built in Adobe Photoshop CS3 (Adobe Systems, San Jose, CA, USA).

### 4.5. Measurement of Infarct Lesion

Mice used for US Doppler imaging were sacrificed 8 days after ischemia. Brains were removed, fixed for 5 days in 4% formol, and then embedded in paraffin and cut into 20-µm-thick sections. Six sections from anterior striatum (0.86 mm) to posterior hippocampus (−2.92 mm) were selected. Brain tissue loss was determined on cresyl violet-stained sections using an image analyzer (ImageJ, National Institutes of Health, Bethesda, MA, USA http://rsb.info.nih.gov/ij/). Total brain tissue loss was expressed in percentage as (1- (ipsilateral hemisphere area/contralateral hemisphere area)) × 100. Area measures were expressed in arbitrary units (A.U.) and obtained at each level of the six sections were reported for each animal.

### 4.6. Statistical Analysis

Doppler ultrasound measurements are reported as mean ± standard-deviation (SD). The Gaussian distribution was assessed using the D’Agostino–Pearson test in all groups. Mean BFVs were analyzed with an ANOVA for repeated measurements (basal–3 h after pMCAo and drug injection) with drugs injected as factor of interaction. Delta-variations and percentage variations in mBFV for each artery and each mouse were calculated and compared between injected drug groups using a one-way ANOVA. Lesion areas were analyzed using a two-way analysis of variance. When ANOVAs were significant, differences between groups were then evaluated with post hoc Bonferonni and paired or unpaired Student t-test (MedCalc^®^ Statistical Software version 18.2.1, MedCalc Software bvba, Ostend, Belgium). The power of the tests was verified and considered significant when >80% (http://www.anastats.fr, ANASTATS, Rilly Sur Vienne, France). Only *p* values < 0.005 were considered significant.

## 5. Conclusions

In conclusion, PJ34 at a single dose was able to induce the recruitment of pre-existing cortical anastomoses in pup mice to preserve the ischemic hemisphere, particularly at the rostral extremity, downstream from a permanent MCA occlusion. This collateral supply and the additional neuroprotective effects of the molecule PJ34 were only sufficient to reduce tissue damage in the rostral extremity.

## Data Availability

The data presented in this study are available on request from the corresponding author in reasonable proportions.

## Supplementary Materials

The following are available online at https://www.mdpi.com/article/10.3390/cimb43010025/s1, Table S1—Absolute values of mBFV recorded in the 3 great arteries of the Circle of Willis, Figure S1—Delineation of SST2 internalization, Figure S2—GFAP-positive astrocytes in the caudal sections after PBS and/or PJ34 treatment.
