# Supplementary material for: Poly(ADP-Ribose) Polymerase Inhibitor PJ34 Reduces Brain Damage after Stroke in the Neonatal Mouse Brain
Source: Curr Issues Mol Biol. 2021 Jun 7;43(1):301–12. doi: 10.3390/cimb43010025 (PMC8929080; doi:10.3390/cimb43010025)
Supplement: Supplementary file 1 [file cimb-43-00025-s001.zip › cimb-1193256-supplementary.pdf]

Online-only material

Poly(ADP-ribose) polymerase inhibitor PJ34 reduces anterior brain damage after stroke in the neonatal mouse brain

Philippe Bonnin, Tania Vitalis, Lesle Schwendimann, Alexandre Boutigny, Nassim Mohamedi, Valérie C Besson, Christiane Charriaut-Marlangue

Results – Supplemental Results and Figures

Supplemental Table 1: Absolute values of mBFV recorded in the right and left internal carotid arteries (ICA) and in the basilar trunk (BT) before and after 3 hours recovery from a permanent distal left middle cerebral artery occlusion (pMCAo).

		Left ICA (cm/s)	Right ICA (cm/s)	Basilar Trunk (cm/s)
PBS	**Basal**	5.2±1.0	5.9±0.9	2.8±0.7
	(pMCAo vs basal)	*P=0.0003*	*P=0.1411*	*P=0.7464*
	**3h after pMCAo**	4.3±0.8	5.3±1.2	2.9±0.6
PJ34	**Basal**	5.6±1.2	5.3±1.7	2.7±0.5
	(PJ34 vs PBS)	*P=0.4996*	*P=0.3630*	*P=0.5346*
	(pMCAo vs basal)	*P=0.0714*	*P=0.0038*	*P=0.0994*
	**3h after pMCAo**	6.0±1.4	6.3±2.0	3.0±0.8
	(PJ34 vs PBS)	*P=0.0046*	*P=0.2249*	*P=0.7453*

Online-only material

Delineation of the region of SST2 internalization

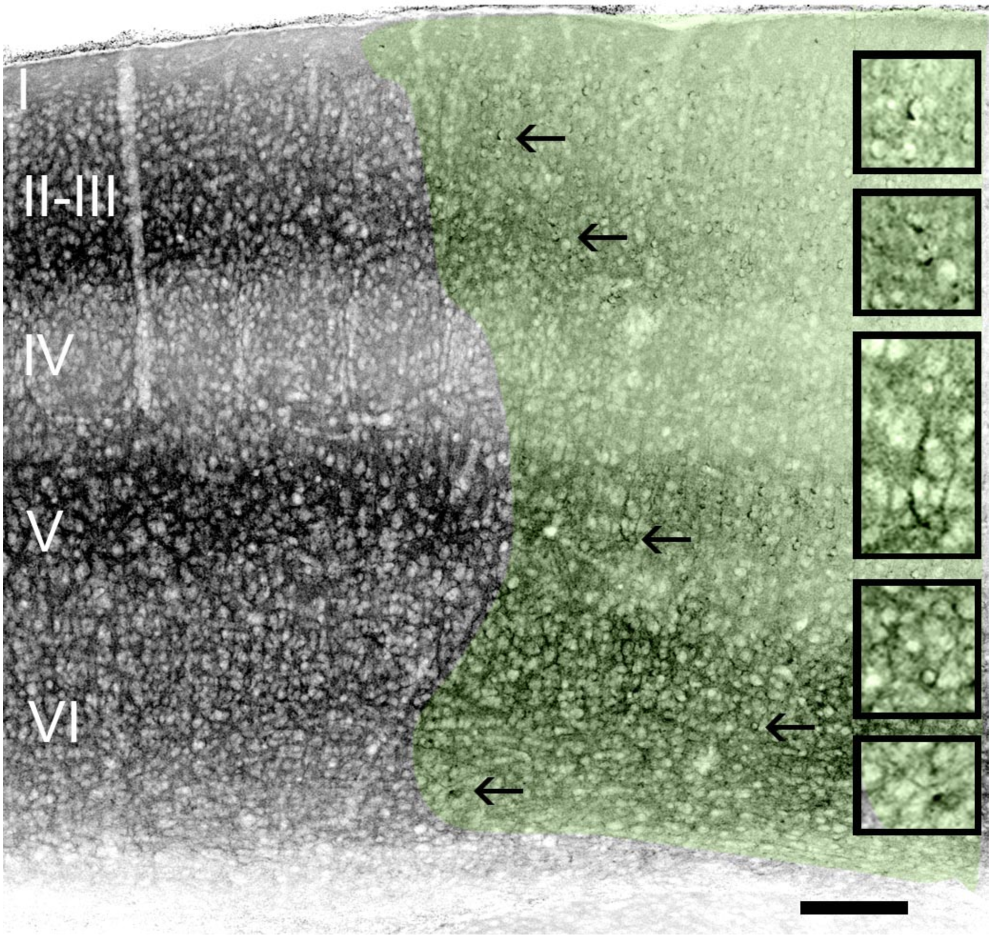

Figure 1: Example of the delineation of the region where SST2 is internalized in octreotide-injected mice following pMCAo. This example is taken at Bregma -1,34 mm in the primary somatosensory cortex (barrels could be detected in layer IV). The caption is taken away from the lesioned area. In the left side of the picture where BBB is intact, SST2 immunoreactivity is homogenously distributed in all cortical layers as SST2 immunoreactivity (a G protein-coupled receptor) is localized at the cell surface of neurons and in a network of dendritic processes. SST2 labeling appears however less intense in layers IV and I, but SST2 distribution is similar to what is observed in other cortical layers. In the region of BBB leakage (colorized area), the somatostatin receptor agonist, octreotide has flown outside the blood vessels and has activated the SST2 receptor. Consequently, activated receptors have internalized from the membrane into perinuclear compartments allowing unambiguous localization of BBB opening. Thus Clear-cut borders between areas of internalization and areas where internalization did not occur were evident. High magnifications in the insets show typical cells with SST2 internalization. Scale bar: 100 mm for the main caption and 40 µm for inserted high magnifications.

Online-only material

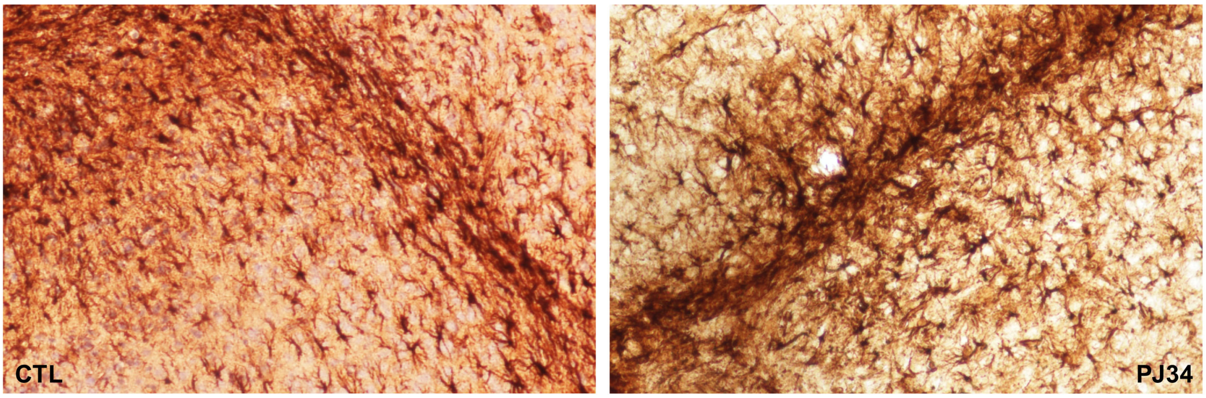

<u>Figure 2</u>: Representative images of reactive astrocytes after PBS and/or PJ34 treatment in the caudal sections (Bregma -1.34 mm) of the brain at 8 days after stroke. Images showed dense GFAP staining in astrocytes and processes and the presence of a glial scar closed to the damaged cortex (images taken at objective x10).
